# Impact of Atraumatic Restorative Treatment (ART) on the treatment profile in pilot government dental clinics in Tanzania

**DOI:** 10.1186/1472-6831-9-14

**Published:** 2009-06-08

**Authors:** Emil Namakuka Kikwilu, Jo Frencken, Jan Mulder

**Affiliations:** 1 Department of Preventive and Community Dentistry, School of Dentistry, Muhimbili University of Health and Allied Sciences, P.O. Box 65014, Dar es Salaam, Tanzania; 2 Nijmegen International Centre for Oral Health Radboud University Nijmegen Medical Centre, College of Dental Sciences, Nijmegen, The Netherlands; 3 Department of Preventive and Restorative Dentistry, Radboud University Nijmegen Medical Centre, College of Dental Sciences, Nijmegen, The Netherlands

## Abstract

**Background:**

The predominant mode of treatment in government dental clinics in Tanzania has been tooth extraction because the economy could not support the conventional restorative care which depends on expensive equipment, electricity and piped water systems. Atraumatic Restorative Treatment (ART) was perceived as a suitable alternative. A 3.5-year study was designed to document the changes in the treatment profiles ascribed to the systematic introduction of ART in pilot government dental clinics.

**Methods:**

Dental practitioners who were working in 13 government dental clinics underwent a 7-day ART training. Treatment record data on teeth extracted and teeth restored by the conventional and ART approaches were collected from these clinics for the three study periods. The mean percentage of ART restorations to total treatment, ART restorations to total restorations, and total restorations to total treatments rendered were computed. Differences between variables were determined by ANOVA, t-test and Chi-square.

**Results:**

The mean percentage of ART restorations to total treatment rendered was 0.4 (SE = 0.5) and 11.9 (SE = 1.1) during the baseline and second follow-up period respectively (*ANOVA mixed model; P < 0.0001*). The mean percentage of ART restorations to total restorations rendered at baseline and 2^nd ^follow-up period was 8.4% and 88.9% respectively (*ANOVA mixed model; P < 0.0001*). The mean percentage of restorations to total treatment rendered at baseline and 2^nd ^follow-up was 3.9% and 13.0%, respectively (*ANOVA mixed model; P < 0.0001*). Ninety-nine percent of patients were satisfied with ART restorations, 96.6% willing to receive ART restoration again in future, and 94.9% willing to recommend ART treatment to their close relatives.

**Conclusion:**

ART introduction in pilot government dental clinics raised the number of teeth saved by restorative care. Countrywide introduction of the ART approach in Tanzania is recommended.

## Background

By 2005, the oral healthcare in Tanzania was mainly public and was rendered in dental clinics situated in regional and district headquarters. The predominant mode of treatment was tooth extraction [1-4] because the economy could not support conventional restorative care [5,6]. The contribution of restorative care to oral healthcare and to oral health of the population was therefore negligible [1-3,7,8]. In addition, rural residents, who constituted 77% of the total population in Tanzania [9], had to travel long distances to urban centres to seek oral care. This unsatisfactory oral health care had existed since independence in year 1961, and a need to seek for alternative approach of managing dental caries in Tanzania became apparent. The available epidemiological data had shown that most of the carious lesions were single surface lesions located in pits and fissures of molars, which could be managed by simple restorative care [7].

Over the last two decades a preventive and restorative caries-management concept based on minimal intervention dentistry has been developed: the Atraumatic Restorative Treatment (ART) [10]. This employs an approach to treating tooth cavities that uses only hand instruments and subsequent filling of the cleaned cavity and adjacent pits and fissures with an adhesive dental material, usually a high-viscosity glass-ionomer [10]. ART does not rely on electricity and piped water systems, required for conventional restorative caries treatment, that are often unavailable in developing countries. Using ART, dentists can provide restorative care in the dental clinic even if conventional equipment is unavailable or out-of-order, and in outreach situations. Studies have indicated that six-year survival rates of single-surface ART restorations in permanent teeth of children and adolescents range from 67% to 75% which are comparable to those of similar conventional amalgam restorations [11-14]. In outreach situations in 3 Latin American countries the ART approach has proved more cost-effective than conventional dental caries management [15]. ART has also been shown to cause less dental anxiety in patients than does conventional restorative treatment [16,17]. Therefore, it has the potential to reduce the burden of toothache and increase the accessibility of oral care in developing countries. Consequently, WHO adopted ART in 1994 [18], the WHO Africa Office included ART in its strategic oral health policy guidelines 1999–2008 [19], and PAHO recommended the use of ART to manage dental caries in Latin-American countries [15].

Atraumatic Restorative Treatment (ART) was perceived as a suitable alternative or complementary approach to treating dental carious lesions in Tanzania. With ART, dental practitioners could restore teeth in dental clinics even if the dental equipment was out of order. In addition, they could restore teeth in outreach clinics in rural areas therefore making restorative care accessible to the majority of population in rural areas. As from 1994, ART seminars, workshops and demonstrations were conducted to different groups of dental practitioners, and in 2002 ART was incorporated into the Policy Guidelines for Oral Health Services in Tanzania as an essential tool for managing dental caries [20]. Nevertheless, its impact on the treatment profile in different dental clinics was negligible [21].

It was not known why ART was not picking up in Tanzania despite of policy guidelines and ART seminars, workshops and demonstrations. Therefore it was decided to run a pilot project for a systematic introduction of ART in few dental clinics in order to identify facilitating and inhibiting factors for adoption of ART in government dental clinics. Lessons learnt from the pilot project were to be used to facilitate country wide dissemination of ART practice in government dental clinics in Tanzania. The aim of the present study was to assess the impact of ART introduction on the treatment profile in pilot government dental clinics and to assess patients' experiences related to it.

## Methods

### Selection of Dental Clinics

The inclusion requirement for participation in the pilot study was willingness of the health authority to: (1) allocate finances for purchasing glass-ionomer and ART hand instruments for use in dental clinics and (2) allow the dental practitioners to participate in a fulltime 7-day ART training course. This was aimed at overcoming potential barriers to changing professional practice related to hospital administration [22], and based on the importance of involving all stakeholders in the introduction of healthcare innovations [23]. Eventually sixteen clinics met the criteria; 3 in Dar es Salaam, 4 in Morogoro, and 9 in the Tanga Regions. A total of 32 dental practitioners were working in these 16 clinics at the start of the project, and were invited to attend an ART training course. Four of the dental practitioners were dentists, 13 were Assistant Dental Officers (ADO), and 15 were Dental Therapists (DT).

### The ART training course

The course was organised in July 2005, in the Dental Therapist School in Tanga and attended by 30 practitioners in two groups of 16 and 14 participants each. Two of the 32 dental practitioners did not attend: one reported that he was denied permission; the second reported to had been caught up with other duties. It began with discussions about the outcomes of studies covering barriers to restorative care as perceived by dental patients and practitioners, and intentions of practitioners to practice ART in Tanzania [24-26]. These were followed by lectures and pre-clinical and clinical practice of ART, using a training manual [27] and publications.

### Supervisory visits and follow-up meetings

A process evaluation was conducted throughout the duration of the project. Included were several checks, built into the training process to address previously documented failures in the introduction of innovations into clinical practice [22]. Participants were tested on the ART approach before and after the course and interactive methods of learning were used to improve understanding of the course material.

Supervisory visits to individual clinics were conducted twice yearly during the follow-up period. The first aim of these supportive visits was to observe the practitioners using ART to treat patients in their own clinic settings and help them to address any doubts regarding their clinical skills, which had been shown to be among the causes for clinical inertia [28]. The second aim was to identify and address any other barriers related to organisation of the clinics that could affect the smooth introduction of ART. The third was to check through patient records to assess the number of ART restorations that had been made within a given time. Discussions were held with hospital authorities on issues related to the introduction of ART and lobbying for continued support was done when deemed necessary.

A one-day follow-up meeting was held once at the end of each follow-up year in each of the three regions, to allow practitioners to discuss their experiences regarding the introduction of ART in their clinics. Constraints at the regional level were also discussed in these meetings.

### Data collection and management

Written approval for this study was obtained from the Ethical Committee of the Muhimbili University College of Health Sciences (reference MU/RP/AEC/VOL.II/130). The treatment rendered in all pilot dental clinics was recorded on the standard patient record forms used in all clinics. These covered tooth extractions, ART restorations and conventional restorations in both primary and permanent teeth. Only data from 13 pilot dental clinics in which the dental practitioners who attended an ART training course worked throughout the follow-up period were included in this analysis. Three pilot dental clinics had incomplete data because the dental practitioners who had attended an ART training course had been transferred to other clinics. Patients' views on the ART approach were collected during the 2^nd ^follow-up period, using the structured questionnaire (Table 1).

**Table 1:** Distribution of patients by their responses to questions on how they had experienced being treated using the ART approach

**Question**	**Number**	**Percent**
Gender		
▪ Men	111	42.4
▪ Women	151	57.6
		
How did you feel during ART procedure?		
▪ I felt no pain	159	60.7
▪ I felt slight/or pain	103	39.3
		
How satisfied are you with the ART treatment		
▪ Satisfied	260	99.2
▪ Dissatisfied	2	0.8
		
Are you willing to get the same treatment next time if need arises		
▪ Yes	258	98.9
▪ No	3	1.1
		
Are you willing to recommend the same treatment to your family member or friend		
▪ Yes	252	97.3
▪ No	7	2.7
		
*Comparing with tooth extraction, ART is a better treatment		
▪ Agree	200	96.6
▪ Disagree	7	3.4
		
$Comparing with restoration using a drill, ART is a better approach		
▪ Agree	111	94.9
▪ Disagree	6	5.1

* Only those who had tooth extraction before responded to this question.

$ Only patients who had received a traditional restoration before responded to this question

The patient questionnaire data and the monthly summary of dental data for the total 31-month post-ART-training follow-up period and the 12-month pre-ART-training period were entered into two data bases, exported into SAS software version 11 and analysed by an oral statistician.

### Construction of variables

The treatments provided included extractions, restorations using conventional rotary equipment and restorations using the ART approach. A new dependent variable, *total treatment*, was constructed: the sum of extracted teeth + conventionally restored teeth + ART restored teeth. Other dependent variables were: *pct-ART-all*, the percentage of ART restorations to the *total treatment *rendered; *ART-fraction*, the percentage of ART to total teeth restored (ART restorations + conventional restorations); and *pct-totalrest*, the percentage of total restorations to total treatments rendered.

The independent variables were *region *(Dar es Salaam, Morogoro, Tanga), *clinic location *(rural, urban) and *period of study *(baseline, 1^st ^follow-up, 2^nd ^follow-up). Baseline was the 12-months pre-ART-training. The 1^st ^follow-up period was the first 21-months post-ART-training, when all practitioners had returned to their clinics with a set of ART hand instruments and one pack of glass-ionomer, after completing the ART training course. Difficulties in obtaining additional supplies of glass-ionomer and ART hand instruments characterised this period. The 2^nd ^follow-up period covered the last 10-months post-ART-training and was characterised by adequate supplies of glass-ionomer and ART hand instruments.

The responses to the questions regarding patient's views on ART treatment that had more than 2 options (Table 1) were dichotomized as follows: *"I felt pain" *and *"I felt slight pain" *were combined into *"I felt pain"*;*"no pain at all" became "I felt no pain"*; "*satisfied" *and *"slightly satisfied*" were combined into "*satisfied"*, while *"slightly dissatisfied" *and *"dissatisfied" *were combined into *"dissatisfied"*;. *"agree completely" *and *"slightly agree" *were combined into *"agree"*', *"slightly disagree" *and *"disagree completely" *were combined into *"disagree"*.

### Statistical analysis

The mean percentages for *pct-ART-all*, *ART-fraction*, and *pct-totalrest *were calculated. Analysis of Variance was performed to test for statistical differences between dependent and independent variables, whereas the t-test was applied to test for differences between the variables. Statistical significance was set at p < 0.05. Data from three clinics were incomplete and were excluded from analysis. The Chi-square test was applied to detect associations between variables related to patients' views on ART.

## Results

Table 1 summarises the responses from 262 patients to questions on how they had experienced treatment with ART. Sixty percent felt no pain during the ART procedure of cleaning and restoring tooth cavities without local anaesthesia, while 99% were satisfied after having received an ART restoration.

Table 2 summarises the number of teeth treated in 13 pilot dental clinics during the period of study. A total of 96,719 teeth, of which 89% were permanent teeth, were treated during the 43-month study period. The monthly mean number of conventional restorations dropped from 65 at baseline to 33 at the 2^nd ^follow-up period. The monthly mean number of ART restorations, on the other hand, increased from 16 during baseline to 219 during the 2^nd ^follow-up period.

**Table 2:** Total and monthly mean number and standard error (SE) of teeth treated in 13 dental clinics by period of study

	Period of study*		
**Total and monthly mean number of teeth treated**	**Baseline**	**1^**st **^follow-up**	**2^**nd **^follow-up**

**Extractions**			
• Total	21,864	44,050	23,762
• Mean	1,822	2,097	2,376
• Standard Error	41.0	46.0	52.5
			
**Conventional restorations**			
• Total	786	856	332
• Mean	65	40	33
• Standard error	3.1	3.0	2.5
			
**ART restorations**			
• Total	190	2,690	2,189
• Mean	16	128	219
• Standard error	4.5	9.9	10.4

*Baseline period = 1 year period before ART training course;

1^st ^follow-up period = 21 months after ART training course, characterised by interruption in supplies;

2^nd ^follow-up period = 10 months of adequate supplies.

The mean percentage of ART restorations to total treatment rendered in 13 pilot dental clinics by region, clinic location and period of study is shown in Table 3. The overall mean percentage of ART restorations to total treatment rendered was 0.4 (SE = 0.5) during the baseline, and 11.9 (SE = 1.1) during the second follow-up period. The differences were statistically significant in both regions and clinic locations (*ANOVA mixed model; P < 0.001*).

**Table 3:** Mean percentage and Standard Error (SE) of ART restorations to total treatment rendered in 13 dental clinics by region, clinic location and period of study

	**Period of study**		
	**Baseline**	**1^**st **^follow-up**	**2^**nd **^follow-up**
	Mean (SE)	Mean (SE)	Mean (SE)

**Region**			
Dar es Salaam	2.0 (0.5) ^a^	3.1 (0.5) ^b^	6.1 (0.6)^c^
Morogoro	0.0^d^	9.0 (0.8)^e^	11.4 (1.0)^f^
Tanga	0.1 (0.1)^g^	7.2 (0.8)^h^	13.8 (1.8)^i^
Total	0.4 (0.1)	7.1 (0.5)	11.9 (1.1)
**Clinic location**			
Rural	0.0 ^j^	8.2 (0.7)^k^	14.1 (1.6)^l^
Urban	0.9 (0.3) ^m^	5.4 (0.8) ^n^	8.3 (0.9) ^o^
Total	0.4 (0.1)	7.1 (0.5)	11.9 (1.1)

ANOVA mixed model: ^no, ci, lo ^*P *< 0.05; ^mn ^*P *< 0.001; ^de, df, gh, fh, hi, jk, jl, kl, mo ^*P *< 0.0001

Table 4 summarises the mean percentage of ART restorations to total restorations in 13 dental clinics by region, clinic location and period of study. The overall mean percentage of ART restorations to total restorations rendered was 8.4 (SE = 2.2) during the baseline, and 88.9 (SE = 1.8) during the second follow-up period. The differences were statistically significant in both regions and clinic locations (*ANOVA mixed model; P = 0.001*).

**Table 4:** Mean percentage and Standard Error (SE) of ART restorations to total restorations in 13 dental clinics by region, clinic location and period of study

	**Period of study period**		
	**Baseline**	**1^**st **^follow-up**	**2^**nd **^follow-up**
	Mean (SE)	Mean (SE)	Mean (SE)

**Region**			
Dar es Salaam	33.2 (6.9) ^a^	53.5 (4.7) ^b^	76.2 (5.4) ^c^
Morogoro	0.0 ^d^	83.8 (2.7) ^e^	92.3 (2.7) ^f^
Tanga	1.6 (1.6) ^g^	76.9 (3.4) ^h^	90.8 (2.2) ^i^
Total	8.4 (2.2)	75.3 (2.2)	88.9 (1.8)
			
**Clinic location**			
Rural	0.0^j^	81.9 (2.2)^k^	91.1 (2.0)^l^
Urban	21.2 (5.0) ^m^	64.3 (4.3) ^n^	85.2 (3.2) ^o^
Total	8.4 (2.2)	75.3 (2.2)	88.9 (1.8)

ANOVA mixed model ^kl ^*P *= 0.03;^mn, ab, bc, ^*P *< 0.01; ^ac, de, df, gh, gi, jk, jl, mn, mo, no ^*P *< 0.0001

The mean percentage of the total restorations to total treatment rendered in 13 dental clinics by region, clinic location and period of study is summarized in Table 5. The overall mean percentage of all restorations to total treatment rendered was 3.9 (SE = 0.4) during the baseline, and 13.0 (SE = 1.1) during the second follow-up period. The differences were statistically significant in both regions and clinic locations (*ANOVA mixed model; P < 0.001*).

**Table 5:** Mean percentage and standard error (SE) of the total number of restorations to all treatments rendered in 13 dental clinics by region, clinic location and period of study

	**Period of study**		
	**Baseline**	**1^**st **^follow-up**	**2^**nd **^follow-up**
	Mean (SE)	Mean (SE)	Mean (SE)

**Region**			
Dar es Salaam	4.3 (0.5) ^a^	4.9 (0.6) ^b^	7.7 (0.4) ^c^
Morogoro	1.9 (0.4) ^d^	11.0 (0.9) ^e^	11.9 (0.9) ^f^
Tanga	5.0 (0.7) ^g^	8.5 (0.9) ^h^	15.3 (1.9) ^i^
Total	3.9 (0.4)	8.7 (0.6)	13.0 (1.1)
			
**Clinic location**			
Rural	4.7 (0.6)^j^	10.1 (0.8)^k^	15.5 (1.6)^l^
Urban	2.7 (0.4)^m^	6.5 (0.8)^n^	9.2 (0.8)^o^
Total	3.9 (0.4)	8.7 (0.6)	13.0 (1.1)

ANOVA mixed model ^no, lo ^*P *< 0.05; ^mn ^< 0.01; ^gh ^*P *< 0.001; ^de, df, hi, gijk, jl, kl, mo ^*P *< 0.0001

## Discussion

The current ART pilot project was undertaken following negligible impact of ART to restorative care in Tanzania despite of the recommendations of WHO Africa Region [19] and of the Ministry of Health and Social Welfare in Tanzania [20] for its use. Both WHO and the Ministry of Health in Tanzania were convinced that ART would improve the management of dental caries; from mainly tooth extractions to more restorative care. To increase chances for success, the implementation of the ART pilot project was undertaken after investigating and solving the potential barriers to restorative care among dental practitioners and patients and ascertaining the willingness of dental practitioners to practice ART [24-26]. In addition, a built-in process evaluation was instituted, to identify and address any other barriers that might have arisen in the implementation process. The check list of important issues to address in the process evaluation was obtained from the extensive literature review of interventions that have been shown to be consistently effective [29-31], potential barriers to changing clinical practice [32,33], and what works best in developing countries when you want to introduce evidence into practice [34]. This was the first time that the ART approach was introduced into a national oral healthcare system in a systematic manner. Therefore experiences of interventions in health care other than those related to introduction of ART in oral health care were used.

Studies to evaluate the effect of an intervention require a control group. As ART required training in order to practice it, dental practitioners who were not trained in ART could not be expected to practice it, which would have rendered comparison between experimental and control groups meaningless. Therefore, a control group was not included in the present study. The treatment data collected one year before the ART training course were considered the control data for subsequent comparison with the data collected during the follow-up periods. Nevertheless, treatment data from comparable non-pilot clinics were compared with those of the pilot clinics, showing close similarity in number of attendances and number of extractions but in lower number of restorations provided over the study period. Therefore, the increased effect of ART restorations relative to total treatment rendered in pilot clinics is a valid outcome.

The current pilot project did not use the mass media to advocate the availability of the ART approach in pilot clinics; for fear that some clinics could lack the capacity to handle all patients who might demand this service because of inexperience in ART practice. Nevertheless, patients who were treated through the ART approach spread the news to their relatives and neighbours. Therefore, the availability of ART approach became known by increasing numbers of patients who came to pilot clinics demanding the service. In view of the experience gained by practitioners in three years, advertising to the community might now be considered.

The finding that the contribution of ART restorations to total treatment rendered during the second follow-up period, which was accompanied by an adequate supply of glass-ionomer and ART hand instruments, was higher than during the first follow-up period shows the importance of government-ensured availability of a constant supply of these essential items. The contribution of ART restorations to total treatment was higher in the present study than in a provincial oral health service system using ART in South Africa [35]. We ascribe the higher success rate obtained in Tanzania to the structured approach used in introducing ART in Tanzania.

The finding that 60% of patients who were treated through the ART approach reported no pain is in line with the reported 68% among children in a remote village in Mexico [36] and among adolescents in Egypt (63%) [37]. Nevertheless, it was lower than the 80.6% reported among adolescents in Pakistan [16]. These findings correlate well with the finding that the ART restorative approach provokes less anxiety in patients during treatment than the conventional approach does [17].

The reduction in the mean number of conventional restorations during the evaluation period indicates that practitioners favoured ART over conventional restorations. The finding that the majority of the patients were satisfied with the ART approach, and that most of patients who had previously received conventional restorative care preferred ART to the conventional approach, showed patients' acceptance of ART. These two outcomes are important to oral health in Tanzania because restorative care can now be undertaken at lower cost, through wide use of ART instead of conventional restorative care using amalgam, which has been shown to be more expensive than ART [15]. Together with preventive and promotional activities, oral health can now be adequately provided to children through the school education system. The systematic approach taken was new and deserves to be applied in developing countries with similar inadequate oral healthcare services as in Tanzania.

## Conclusion

The introduction of ART in pilot government dental clinics raised the number of teeth that were saved by restorative care, which otherwise could have been extracted. Patients appreciated the ART approach. Introduction of ART to other regions in Tanzania, and to developing countries, under close monitoring, is recommended.

## Competing interests

The authors declare that they have no competing interests.

## Authors' contributions

EK participated in conception and design of the study, training practitioners, follow-up supervision, and data collection and drafting the manuscript. JF participated in conception and design of the study, training practitioners, guidance in the management of the pilot study, review of manuscript. JM participated in the study design, statistical analysis and reviewed the manuscript. All authors read and approved the final manuscript.

## Pre-publication history

The pre-publication history for this paper can be accessed here:


